# Service-users’ perspectives of link worker social prescribing: a qualitative follow-up study

**DOI:** 10.1186/s12889-018-6349-x

**Published:** 2019-01-22

**Authors:** Josephine M. Wildman, Suzanne Moffatt, Mel Steer, Kirsty Laing, Linda Penn, Nicola O’Brien

**Affiliations:** 1 Institute of Health & Society, Newcastle University, Newcastle upon Tyne, UK; 2 Newcastle Institute for Social Renewal, Newcastle University, Newcastle upon Tyne, UK

## Abstract

**Background:**

Social prescribing enables health-care professionals to address non-medical causes of ill-health by harnessing the resources of the voluntary and community sectors in patient care. Although increasingly popular in the UK, evidence for the effectiveness of social prescribing is inconclusive and longer-term studies are needed. This study aimed to explore experiences of social prescribing among people with long-term conditions one to two years after their initial engagement with a social prescribing service.

**Methods:**

Qualitative methods comprising semi-structured follow-up interviews were conducted with 24 users of a link worker social prescribing service who had participated in an earlier study. Participants were aged between 40 and 74 years and were living in a socioeconomically-deprived area of North East England.

**Results:**

Participants reported reduced social isolation and improvements in their condition management and health-related behaviours. However, many participants had experienced setbacks, requiring continued support to overcome problems due to multi-morbidity, family circumstances and social, economic or cultural factors. Findings indicated that, in this sample of people facing complex health and socioeconomic issues, longer-term intervention and support was required. Features of the link worker social prescribing intervention that were positively appraised by participants, included a highly personalised service to reflect individual goal setting priorities and a focus on gradual and holistic change dealing with issues beyond health. The important role of a strong and supportive relationship with an easily-accessible link worker in promoting sustained behaviour change highlights the importance of link worker continuity. A lack of suitable and accessible voluntary and community services for onward referral acted as a barrier to involvement for some participants.

**Conclusions:**

This study highlights issues of interest to commissioners and providers of social prescribing. Engagement with social prescribing for up to two years was examined and continued involvement was identified for those with complex issues, suggesting that a long-term intervention is required. The availability of onward referral services is an important consideration for social prescribing in a time of constrained public spending. From a research perspective, the range of improvements and their episodic nature suggest that the evaluation of social prescribing interventions requires both quantitative and qualitative data collected longitudinally.

## Background

Managing the increasing prevalence of long-term conditions (LTCs) is one of the greatest challenges facing healthcare systems [1] and, for the past decade, helping people with LTCs ‘take control’ of managing their health has been a focus of the UK Government and the National Health Service (NHS) [2, 3]. Developed with the aim of encouraging self-care and behaviour change, ‘social prescribing’ interventions (sometimes called ‘community referral interventions’) allow health-care practitioners to refer patients with LTCs to non-clinical services, primarily in the community and voluntary sectors. Health behaviour change can be difficult to achieve without support and simply ‘signposting’ to sources of community support is unlikely to be successful [4]. Therefore, to increase service-user engagement, most social prescribing schemes involve a ‘link worker’ (alternative titles include ‘social prescribing co-ordinator’, ‘health trainer’ and ‘community navigator’ [5]) who provides personalised support and helps service users to access sources of support within their community [6, 7].

Social prescribing interventions are often targeted at people in socioeconomically deprived areas, expanding options available to primary-care practitioners when patients present with needs related to wider social determinants of health [8]. Social, rather than health, problems place considerable burdens on primary care, with 20% of patients consulting their general practitioner (GP) for primarily social problems and 15% of patients visiting for welfare-benefits advice [9]. A common criticism of public health interventions is their tendency to focus on individual-level health behaviours and overlook the structural determinants of health [10]. ‘Holistic’ social prescribing interventions seek to address the wider social determinants of health and, therefore, go beyond the neoliberal standpoint of viewing individual health behaviours as the personal failings of freely-choosing individuals [10]. However, a major element of social prescribing remains individual behaviour change through ‘empowerment’ of service users to make better choices [11].

In the UK, there is increasing interest in the potential of social prescribing to address issues associated with chronic ill health [3, 5, 7]. Self-care and social prescribing programmes are part of NHS England’s ‘Five Year Forward View’, which aims to promote healthy communities and support people with LTCs [12]. Social prescribing is also a key plank of the ‘New Deal for General Practice’ strategy [13]. However, despite its growing prominence, to-date the evidence base for the effectiveness of social prescribing is inconclusive [14] and a need has been identified for studies of longer-term effects [7, 15]. The current qualitative study aims to explore service-users’ experiences of social prescribing, one to two years after their initial involvement with ‘Ways to Wellness’, a link worker social prescribing intervention. We investigate factors enabling engagement and encouraging behaviour change and the extent to which social prescribing was successful in a group facing a range of structural barriers to improving their health behaviours and LTC management.

### ‘Ways to Wellness’ social prescribing intervention

Ways to Wellness (WtW) has been delivering link worker social prescribing since April 2015. The intervention was developed over an eight-year period, following extensive consultation with both patients and health-care professionals [16]. Ways to Wellness serves 17 General Practices in west Newcastle upon Tyne (UK), an inner-city area of socioeconomic deprivation. Practices refer to the intervention  patients aged between 40 and 74 with one (or more) of the following LTCs: diabetes types 1 and 2, chronic obstructive pulmonary disease, asthma, coronary heart disease, heart failure, epilepsy, and osteoporosis, with or without anxiety and/or depression. In the WtW model, patients are referred by a primary-care practitioner to a link worker. Link workers are trained in behaviour change methods, such as motivational interviewing techniques, that help service users identify which areas of their lives they wish to change and how. These techniques emphasis service users' choice and control over their decisions and behaviours [17]. The link worker contacts the patient by telephone to arrange an initial appointment. This could be at the GP practice, a community hub or, less often, at a patient’s home. Link workers help service users to identify personalised and achievable goals. At the initial appointment and every six months thereafter for the duration of their engagement with the intervention, service users complete a ‘Wellbeing Star’ ™, a proprietary tool which identifies target areas for improvement and monitors service-users progress towards their goals. Ways to Wellness is a holistic social prescribing intervention and as well as covering self-care and symptom management, the ‘Wellbeing Star’ ™ also addresses relationships, housing, debt, welfare benefits, and work or volunteering. Service users are supported by their link worker to access appropriate services and community groups (e.g. weight-management groups, welfare rights advice and arts-based activities), and to return to work or engage in volunteering opportunities (as appropriate). Service users remain with the intervention for up to two years or, with link worker discretion, longer if required. Over the course of a patient’s engagement with WtW, face-to-face contact is also supplemented by telephone, email or text contact. Meeting duration frequency increases or decreases according to need. Support offered through WtW can be intensive. For example, if necessary link workers will accompany service users to community activities or assist with completing welfare benefit applications.

## Method

### Population and setting

The setting for this study was an ethnically diverse inner-city area in the west of Newcastle upon Tyne (population *n* = 132,000) ranked among the 40 most socioeconomically deprived areas in England [18]. A higher-than-average proportion of the west Newcastle population have LTCs and are in receipt of sickness or disability-related benefits [19].

### Data collection

Thirty WtW service-users, who had been identified using maximum variation sampling to ensure variation along lines of age, gender and ethnicity and who had participated in an earlier interview study [20], were re-contacted for this study. Twenty-four agreed to a second interview (of the six participants lost to follow-up, two withdrew from the study and four could not be contacted). A topic guide was developed covering: significant life events since last interview; current involvement with WtW and current views on the service; progress with behaviour change; role of the link worker in behaviour change maintenance; and future involvement with WtW. A semi-structured interview with each participant was conducted between November 2016 and April 2017. At the time of interview, participants had been involved with WtW for between 12 and 24 months. Interviews took place at participants’ homes and were conducted by MS and KL. Ethical approval for the study was obtained from Newcastle University Faculty of Medical Sciences Research Ethics Committee. Participants were given written information about the study, were informed that participation was voluntary, that they could withdraw at any time and that confidentiality was assured. Informed written consent was obtained.

### Transcription, data management and analysis

This study adopted a ‘grounded theory’ approach [21, 22] in that, rather than being guided by a particular theoretical perspective, it was led by service-users’ perspectives and focused on emergent narratives. Interviews lasted between 11 minutes and one-hour and 20 minutes (average length 48 minutes) and were digitally audio recorded and transcribed verbatim. Transcripts were anonymised and checked against recordings for accuracy. Thematic analysis was used [23] with data management supported by NVivo 10 Software [24]. Following close reading of the transcripts, a coding scheme was developed containing a-priori themes based on the topic guide and further themes that emerged from the data. The coding scheme was initially applied to three randomly-selected transcripts, which were independently double-coded by JMW and LP. The scheme was then reviewed and agreed modifications were made before applying the scheme to all the interviews. Line-by-line coding and constant comparison were used to code the entire dataset [22, 25]. Deviant cases, where opinions modified or contradicted the analysis, were identified to enhance validity [26].

## Results

### Participant characteristics

The sample comprised 24 participants, 11 women and 13 men, aged 40–74 years. Table 1 reports participants’ characteristics. Sixteen participants described experiencing mental health and social isolation issues. Levels of multi-morbidity were high, with all-but-one participant reporting multiple LTCs. Results from the data analysis revealed four key themes: 1) the importance of service users’ relationships with the link workers who helped them access and navigate community services; 2) factors involved in making and maintaining progress in behaviour change and LTC management; 3) setbacks and barriers to maintaining change; and 4) fluctuating levels of engagement with the intervention.

**Table 1:** Participant characteristics

ID	Sex	Age-band	Employment status	No. of WtW LTCs^a^	No. of non-WtW conditions^b^	Mental health /social isolation^c^	Months since commenced WTW involvement	Current involvement with link worker
*1*	*Male*	*70–74*	*Retired*	*3*	*3*	*Yes*	*N/a*	*N/a*
2	Female	70–74	Retired	3	3	Yes	17	Health problem preventing involvement
3	Female	45–49	Employed	1	1	Yes	14	Involvement decreasing in intensity as situation improves
4	Female	55–59	Unemployed	2	1	Yes	14	LW changed twice. No contact from new LW
5	Female	65–69	Retired	2	4	Yes	15	Involvement decreasing in intensity as situation improves
6	Male	65–69	Retired	2	1	Yes	16	Same level of link worker contact
7	Male	55–59	Unemployed	2	1	No	21	No contact from new LW
8	Female	55–59	Unemployed	2	3	No	16	Involvement decreasing in intensity as situation improves
*9*	*Male*	*55–59*	*Employed*	*3*	*3*	*Yes*	*N/a*	*N/a*
10	Male	65–69	Unemployed	2	4	Yes	17	Health preventing involvement
11	Male	45–49	Unemployed	2	4	Yes	14	No contact from LW
12	Male	55–59	Unemployed	1	3	Yes	12	Involvement decreasing in intensity as situation improves
13	Male	60–64	Unemployed	1	2	Yes	12	No contact from new LW
*14*	*Male*	*60–64*	*Unemployed*	*2*	*1*	*No*	*N/a*	*N/a*
15	Female	40–44	Unemployed	1	0	No	19	Involvement decreased after link worker change
16	Female	65–69	Retired	2	0	No	18	Same level of link worker contact
17	Female	50–54	Unemployed	2	0	Yes	15	Involvement decreasing in intensity as situation improves
18	Female	65–69	Retired	3	2	Yes	16	Involvement decreasing in intensity as situation improves
19	Male	65–69	Retired	2	4	Yes	16	Less involvement due to health problem
20	Male	70–74	Retired	2	1	No	17	No contact for a few months as felt he had gained maximum benefit
21	Female	70–74	Retired	3	1	No	18	Same level of contact with LW
*22*	*Female*	*40–44*	*Unemployed*	*2*	*2*	*Yes*	*N/a*	*N/a*
23	Male	70–74	Retired	2	4	No	22	Less contact due to health problem
*24*	*Female*	*70–74*	*Retired*	*2*	*0*	*Yes*	*N/a*	*N/a*
25	Female	50–54	Unemployed	3	1	Yes	18	Same level of contact
*26*	*Male*	*70–74*	*Retired*	*2*	*3*	*No*	*N/a*	*N/a*
27	Male	40–44	Employed	1	1	Yes	24	No contact from new LW
28	Male	70–74	Retired	2	1	No	24	Less involvement due to a health problem
29	Male	60–64	Employed	1	3	Yes	NK^d^	Less involvement due to change of LW
30	Male	45–49	Unemployed	2	3	Yes	NK^d^	Same level of contact

Italicised text represents participants lost to follow-up

^a^Medical-practitioner diagnosed ‘Ways to Wellness’ referral conditions

^b^Self-reported at interview

^c^Broad category that includes low mood, anxiety, depression, loneliness and social isolation and based on self-report at interview where participants described or reported these conditions or feelings

^d^Not known

### The importance of the service user/link worker relationship

The rationale behind the link-worker role is that without support, navigating and accessing community services can be extremely challenging for some groups of people [4]. For all participants in this study, the link worker was a central figure in their experience of social prescribing and the majority of participants had developed strong relationships with their link worker. Indeed, some described the relationship in terms of friendship:

*I look at him [link worker] as like a pal. It’s as simple as that.* (P19, male, age 65–69)

*She [link worker] was very friendly…She was there to just, generally, talk to. Like, a female companion type thing, because I've got none of that at home, it’s all males. Then it’s just like, I don't know, she’s just so friendly. We used to have a laugh, I would talk about my family, she would talk about hers. It wasn't as though she was like a worker, you know what I mean? It was that good. (P4, female, age 55–59)*

Link workers’ non-judgemental attitudes were highlighted as important for developing a trusting relationship:

*They make you feel normal, that it’s just not your fault. Whatever you’re feeling is fine. Whatever you say is fine. (P17, female, age 50–54)*

A lack of self-confidence was widely reported (both in this current study and in the earlier study [20]) by participants and some recalled their concerns at the start of their involvement with social prescribing. Service users described how the link worker had played an important role in introducing them to new, beneficial activities and services they would otherwise have avoided. Sustained engagement with the intervention led to improved self-esteem and increased confidence around attending their referral activities:

*...when I first went and I was talking to my link worker and I said to them, “I’m not very comfortable about coming into a gym.” He said, “Well shall we talk through what you're not comfortable about?” I said, “Well if I'm going to be going in there, my age, my shape and I'm going to find leotards and skinny minnies in there, I think I'll feel as though I don't fit in at all.” He said, “I can assure you that that will not be the case.” Do you know, it wasn’t? I went there initially not feeling very confident and then, over a very short period of time, I was confident. So it’s boosted my confidence as well. I never saw a leotard! (P2, female, age 70–74)*

Participants favourably contrasted their relationship and interaction with their link worker to their interactions with healthcare professionals, which were often characterised as impersonal and too rushed to properly address the breadth of their social problems.

*There’s a huge difference [between a link worker and a nurse or doctor]. The practice nurse just wants to stick the jab in your arm, and let them get on with it, and that’s it. Doesn’t ever really have time to do the in-depth analysis of where you’re at and what you’re doing. The Ways to Wellness person has that concern. I don’t want people going home and having sleepless nights over me, but it’s nice to think that they do care, and I really feel that they do. (P18, female, age 65–69)*

Link workers’ wide knowledge of the range of community services was also valued as separate to the specialist, health-focused knowledge of health-care professionals.

*…it’s important to have someone there, who has a finger on the pulse, knows all these different things. Doctors can’t know everything and I mean, what they know obviously helps improve your health, but things like support in the community and things, I don’t think enough of them know about it. I don’t even know that the practice nurses know enough about it. (P8, female, age 55–59)*

The holistic nature of the intervention and the presence of someone who “puts all the links together, which is a link worker” (P2, female, age 70–74) in an intervention where “everything was involved” (P29, male, age 60–64) were considered particularly helpful in making lasting changes. As adults living with LTCs in an area of socioeconomic deprivation, many participants had problems beyond LTC management alone and reported receiving support from their link worker across a number of areas, including housing, debt and welfare benefits:

*“They've helped me, sorted my finances and that out and they helped me with getting in touch with certain groups of people on my finances, which I was worried about at the time. That’s getting sorted. That got sorted. They helped me in a lot of different ways because I thought I was losing my mind and that, but I think I'm getting a bit better.”* (P13, male, age 60–64)

### Making and maintaining progress in behaviour change and LTC self-management

#### Nature of behaviour change and LTC self-management

The range of self-reported behaviour changes reported in the earlier study [20] were also reported at follow-up and comprised achieving and maintaining positive changes in diet, physical activity and smoking cessation; improvements in mental health and self-confidence; decreased social isolation; and increased engagement in community activities. The chronic nature of LTCs meant that self-reports of improvements in physical health were rare. However, participants noted that their confidence and ability to self-manage LTCs had improved. Many participants felt confident they could continue with the coping strategies and changes they had made earlier in their engagement with the intervention, or at least were growing in confidence now that they were “better at putting myself right” (P18, female, age 65–69).

#### Factors associated with making and maintaining progress in behaviour change and LTC self-management

A range of factors were associated with successful sustained behaviour change and LTC self-management. A combination of accessing specialist health support services and conversations with their link worker encouraged participants to reflect on their health conditions. This resulted in increased understanding and awareness of their health and improved their coping skills:

*It’s opened me up a lot more. I mean, I start thinking about my health, where I didn’t in the past…it’s a good thing to have this ‘Ways to Wellness’. I think people need it. It’s made me aware.* (P5, female, age 65–69)

*The mood is still the same, because I know the diabetes does cause the moods, but I understand it now and I know what’s going on, so you just pull yourself out of it, as such.* (P6, male, age 65–69)

The WtW intervention is a two-year programme that encourages long-term behaviour change. The emphasis on gradual change was identified as particularly valuable, enabling the setting of “mini-goals” that represented “achievable somethings” (P8, female, age 55–59) in order to make progress towards goals such as a return to employment. Continued self-regulation was required to maintain changes. Many participants had an understanding that LTC management was life-long and the battle was never completely won:

*You can’t [stop making health improvements]. You really, really can’t because then it’s the slippery, slippery slope back down. I mean, yes okay, a day or two out of it, that’s okay, that’s doable. Everyone has days when they’re not too great, but you couldn’t stop completely. If you let it slide, as I say, then you get on the spirally slope downwards where the less you do, the less you feel like doing anymore. You know, your condition deteriorates so you can’t get back to the bad old ways. (P8, female, age 55–59)*

There was an almost universal belief among participants that willpower was vital in maintaining changes over the long term. While a link worker could “encourage and support” (P5, female, age 65–69), long-term change was about “taking responsibility for yourself…nobody else is going to do it” (P18, female, age 65–69). The longer-term nature of the intervention was identified as important for incorporating changes into routine practice so that they became, “automatic…a habit. It’s second nature now” (P6, male, age 65–69).

Participants were ‘linked’ to a wide range of community groups and services, including gyms, walking groups and exercise classes; weight-loss and healthy eating groups; and LTC management groups such as breathing exercises for people living with respiratory conditions. Interviews identified sources of motivation for continued involvement with these activities. Experiencing improvements and successes, such as increasing fitness and weight-loss, proved powerful intrinsic motivators to maintaining change for some, while validation from friends or family were extrinsic sources of motivation for others. For socially isolated participants, increased social contact and the chance to make friends with people in a similar situation was a motivating factor for continued involvement:

*Even the people that went were all in the same boat as me: all overweight, or most of them were. It was just nice. You got to speak every time. Or if somebody was new coming in, they would come by and say, “Fresh meat for the slaughter!” (Laughter) as you do. Anyway, no, everything’s great. I love the gym. (P19, male, age 65–69)*

*So you get to know people every week you're there [community group]…I have to come or they will keep saying, “Are you coming next week? Are you doing this? Are you involved?” So other people are asking me will I be there. The friends that I've taken or whatever. (P7, male, age 55–59)*

### Setbacks and barriers to making and maintaining change

Although some participants were adamant that the changes they made “were actually permanent…we won’t go back to that now” (P2, female, age 70–74), uninterrupted trajectories of improvement were not a universal experience. Maintaining self-regulation is challenging when psychological and physical resources are low, making relapse likely [27]. Echoing participants’ perceptions of the importance of willpower, maintaining sustained behaviour change was widely acknowledge as being “very, very hard” (P10, male, age 65–69). Unsurprisingly in a population with complex and multiple health problems, the most commonly-reported setbacks were health-related. For five participants, a health problem had resulted in reduced engagement with their link worker (Table 1). Some participants reported setbacks resulting from interactions between LTC symptoms or treatment for multi-morbidity that complicated behaviour change maintenance:

*I haven't been to the gym since I got the blood clots. I can't go, not yet, anyway… I'm just waiting for them to tell me when I can get back to normal. Because I haven't been to [the gym] for a fortnight, since I had pneumonia. I'm hoping to get back this week… I went onto Warfarin, which I had to go on for the blood clots, I couldn't eat green vegetables. They took me off them. So you really can't stick to the plate [diet advice] thing. (P23, male, age 70–74)*

Unanticipated health shocks or trauma could impact on progress and this could be demoralising:

*I had quite a nasty fall…I learnt, after an MRI scan, that I'd torn the ligament in my left knee. At the time when I did it, I could barely walk, let alone anything else…I was still going to the gym but since then, that is why I haven't been…So in some respects I feel as though the whole lot has just went down the drain now. All of my hard work, all of their input, through no fault of mine it’s all just been swept away. (P2, female, age 70–74)*

Also reported was the psychological burden of living with LTCs, which could create barriers to progress that could be as strong, or stronger, than the physical impact of a condition:

*I just get paranoid when I get far from that area where I live, for example, so…because I always have pain, still have pain on the chest always, so it made me a little bit paranoid. (P27, male, age 40–44)*

For participants with depression and anxiety, motivation to try new groups and activities could be a particular problem:

*As I say, I just haven’t got the energy, not at all. You get the days where you are feeling dead down and you can’t be bothered. That is the way I feel at the minute…she [link worker] has asked me to join groups and different things. There are things I want to do, but over these last few months I just haven’t had the energy, I just didn’t want to go or even mix with people. (P10, male, age 65–69)*

The appropriateness of onward referrals to voluntary and community groups was also important for continued engagement with the intervention. Participants were generally positive about the groups and services to which they had been referred. However, certain aspects presented physical barriers to engagement with activities including lengthy and costly travel, unsuitable scheduling (for example, after dark or during working hours) and/or a location in an area considered unsafe. Some younger participants identified age as a barrier, with many activities being aimed at older people. Black and Minority Ethnic participants identified further obstacles to taking up referrals, including language barriers, a lack of women-only exercise sessions and cultural appropriateness. One such participant reported that the healthy eating advice offered was unsuitable due to the difficulties of adapting her family’s diet to Westernised norms of ‘healthy eating’ recommended for people with type 2 diabetes:

*The recipes she gave us were the type of food we wouldn’t have eaten anyway. We’ve realised we can’t change our food. We can’t. I’ve tried. I will lose weight if I eat meals like an English person eats. They have a plate and have boiled veg, potatoes and a protein…we can’t eat food like how you have boiled potatoes, veg and protein. I’ve tried that, but then my husband won’t eat that…I will lose weight if I eat the English food, but then when I want to eat the other food, which I crave, it comes back. So, now what I do is I just eat that food…It’s the taste we’re used to. We can’t eat bland stuff all the time. I’ve tried it on him. It didn’t work. He’d eat it and then go, “I miss it. I feel miserable.” He hates it. So, we’ve realised that, and my mum has realised that, and I’ve realised that doesn’t work. (P15, female, age 40–44)*

Home and family environment presented additional barriers to maintaining behaviour change for some participants:

*I can't give up [smoking]. I can go out and about and not smoke, but when I go home, it’s like a smoking atmosphere. I mean, at home, I'm looking at my watch, thinking, “What time can I have my next smoke?” …There’s a lot more smoking going on in the house. I'm not doing it, but I've got my husband and two of my sons and one of them smokes like a trucker. It’s literally one after the other. So, I don't think that’s helping it. (P4, female, 55–59)*

In the previous study [20], participants had been universally positive about their experience of link worker social prescribing. This follow-up study demonstrated that as participants progressed through the programme, most remained positive. Indeed, some described the experience as transformative:

*I think it’s changed my life completely…I was too fat. I had issues. Ways to Wellness has swept some of them away. It’s been a very, very positive experience...I'm a happy bunny. (P19, male, age 65–69)*

However, over the period of engagement, some participants expressed negative views primarily due to personnel changes among link workers that resulted in lost continuity. These accounts highlighted the importance of the link worker/service user relationship and how changes to this highly-valued and often therapeutic relationship could be upsetting and lead to disengagement:

*Well, I don't get as much support now. My first worker left, I used to see her a lot. I was put onto another one, who I've only seen about two or three times. Now she’s left and they’ve put me onto somebody else who I've never seen or been contacted by. I feel a bit let down because my first one was brilliant. She was on the phone, talking, we used to meet up and it was great. I just feel as though I've been let down now…I just feel as though I've been pushed to one side. I don't know what’s going on with the leaving and stuff like that. I just can't understand why the new one hasn't phoned up to introduce herself to me. (P4, female, 55-59).*

Low self-confidence could make establishing a relationship with a new link worker seem daunting, with a reluctance to instigate contact for fear of being a ‘nuisance’ or of appearing too forward: “I’ll wait until [my new link worker has] contacted me because I think it might be a bit cheeky if I phoned up” (P4, female, age 55–59). While these examples were rare overall, the strength of feeling expressed by those whose change of link worker was not perceived to be well-managed highlights the importance of the link worker to successful outcomes and productive engagement with the intervention.

### Fluctuating levels of engagement with social prescribing

The principle behind social prescribing is that as service users become ‘linked’ back into their communities, the intervention can be withdrawn. At the time of these follow-up interviews, participants had been involved with the intervention for between one and two years and described being at various stages of their ‘journey’. Some participants had maintained a constant level of contact with their link worker, while others had less frequent link worker contact as they progressed. As participants achieved their goals, reaching what one participant described as “maintenance point” (P8, female, age 55–59), they reported a natural decline in frequency of contact with their link worker. These participants felt that, rather than intensive support they simply needed someone to occasionally ‘check-in’ with, while they continued their involvement with the groups and services to which they had been linked:

*It was quite intense when [previous link worker] was first there. I used to go in there for an hour, an hour-and-a-half, going through things…I think, with [previous link worker] at the beginning, we talked about everything, and if something wasn’t right, we would say to each other. This guy now [current link worker] I’ve only met him twice, but everything seems sorted out. All I need is somebody to keep going. You know? (P29, male, age 60–64)*

Involvement with the intervention has a duration of two years unless a link worker feels longer involvement is necessary. Awareness of the intervention as time-limited varied among participants, ranging from a belief that “they’ve got strict time limits” to a belief that “it’s as long as I need them, that’s it”. While some participants felt they would be content with two years’ contact, others facing particular challenges (for example, poor mental health or homelessness) wished to continue long-term with the social prescribing programme, feeling “I will always need somebody to help me” (P16, female age 65–69). The fluctuating and chronic nature of LTCs resulted in an almost universal feeling that the opportunity to re-contact their link worker and, if needed, re-enter the programme “for a second bite of the cherry” (P18, female, age 65–69) would be desirable:

*I mean they can extend it. I mean it depends what people’s needs are at the time I suppose. I mean with me, I’d still want to be in contact somewhere along the line, which I think they will do. If something happened to me, if I had an angina attack or something I think I would need them full time all the time then. I know if I have a bad angina attack I’m not going to recover that well. (P12, male, age 55–59)*

## Discussion

This study provides longer-term follow-up qualitative data on experiences of a link worker social prescribing programme one to two-years after initial engagement with the service among people with LTCs living in a socioeconomically disadvantaged region in North East England. Participants reported continuing to make and/or build on earlier self-identified improvements. Further, self-reported improvements were described across a range of areas in and beyond health, supporting research indicating that successful behaviour change interventions are often holistic, dealing with problems in more than one domain [28].

This study suggests that ‘ingredients’ likely to be necessary for continued involvement with social prescribing include an individualised intervention, access to suitable onward referral activities and a focus on gradual behaviour change over period of time (in the case of the WtW service, this period is up to two years). Many participants had also experienced setbacks and needed continued support to overcome problems due to multi-morbidity, family circumstances and social, economic or cultural factors.

It has been suggested that ‘linkage’ to onward referrals via a link worker underpins successful social prescribing [29, 30]. This study supports earlier findings [7, 20] link worker/link worker relationship is vital for participants’ continued engagement with social prescribing programmes. While onward referral groups and services were helping participants achieve their goals and providing valued social contact, the service user/link worker relationship was central to helping service users overcome barriers to accessing and navigating community resources. Indeed, the link worker qualities valued by WtW participants are an excellent fit with Brandling and House’s ([31]:pg.15) description of the ‘ideal’ link worker as “someone with highly developed interpersonal communication and networking skills, with a motivating and inspiring manner to encourage clients to make brave decisions or take up new opportunities”. The types of support offered by link workers in this intervention are similar to those identified in a recent study of lay health workers [32]. This included the ‘emotional’ support and “everyday reassurance” found to be important in supporting service users lacking self-esteem and experiencing anxiety; ‘instrumental support’ (e.g., with filling out welfare benefit application forms); ‘informational’ support in identifying sources of help within the wider community; and ‘appraisal’ support with decision-making and problem-solving ([32]:pg.101). Findings from our study suggest that intensive support provided by link workers is likely to be a more successful model of social prescribing than simply ‘signposting’ to community resources. In addition, the strength of the service-user/link worker relationship identified here suggests that when continuity with a link worker is not possible, expectation management and careful management of the change is likely to be important for keeping service users engaged in the programme.

The WtW social prescribing programme is time-limited but generously so in relation to other similar programmes. There was a sense among many participants that they had travelled a long way since their initial involvement and, as they felt better able to cope, their need for support was naturally decreasing. However, other participants felt their need for support was undiminished, reinforcing a previously identified need for interventions that provide ongoing support for those with more complex difficulties [33]. The need for long-term support coupled with the strength of some service users’ relationships with their link worker (characterised by some as a ‘friendship’) raises the possibility of service-user dependency. Support for people beyond the life of an intervention is likely to be an important aspect of link worker social prescribing, with clear pathways identified for those who need longer to benefit, experience a change in circumstances or who require support beyond the duration of the programme.

By addressing wider social determinants of health and person-centred behaviour change, we would argue that social prescribing attempts to move beyond the neoliberal advocacy of personal responsibility as the sole focus for interventions [10]. However, individual ‘empowerment’ still remains central in many interventions [11]. This study identifies service users’ belief in the central role of self-regulation and ‘willpower’ in behaviour change. This is similar to the concept of ‘no legitimate dependency’, where individuals assume almost complete personal responsibility for managing their own health despite the existence of considerable structural barriers that limit the potential for self-improvement ([34]:pg.174). Self-regulation of behaviour has been found to be only of limited success in previous public health interventions [35]. Long-term behaviour change requires lowered ‘opportunity costs’ through environmental changes that make making better choices much easier [27]. To this end, public health interventions should also seek to improve ‘choice architecture’ in socioeconomically-deprived areas (e.g. improved access to healthy food or better access to green spaces) [27].

This study supports previous findings that highlight the importance for successful social prescribing of good-quality community groups and services for onward referral [30]. Link workers attempt to ‘empower’ service users to address their own problems by accessing sources of support within their communities and social prescribng is therefore highly dependent on good quality public services. A recent study conducted with link workers found that they were placed under considerable stress by attempting to fill the gap left by the withdrawal or severe reduction in support services such as welfare and housing advice [36]. The UK government’s continued programme of economic austerity, resulting in reduced public and community services, is likely to threaten the potential improvements that social prescribing interventions can achieve [31, 37].

### Limitations

Although maximum variation sampling was used to identify a wide range of participants for the earlier study, six participants were lost to follow-up. However, the remaining 24 participants represent the full range of characteristics in the original sample (see Table 1). Further, we cannot be certain of the extent to which the experiences of participants in this study reflect those of all service users nor make any claims about the experiences of people who did not engage or engaged only briefly with the intervention. Finally, improvements arising from social prescribing may be periodic, making them difficult to capture and measure reliably. A ‘methodologically flexible’ approach to assessing the impact of social prescribing has been advocated [7]. Future evaluations of social prescribing should aim to use a range of outcomes (capturing potential benefits to the individual and wider beneficiaries, including family and wider social networks) and a mixed-methods approach utilising both qualitative and quantitative data collected longitudinally.

## Conclusion

The healthcare system is under pressure from the increasing prevalence of LTCs. As an intervention for tackling complex psychosocial and physical health problems among people with LTCs living in socioeconomically deprived areas, social prescribing is continuing to grow in popularity among policy makers, health-care practitioners and commissioners in the UK. This is reflected in a recent announcement of £4.5 million of funding to support the implementation of social prescribing schemes in England [38]. This study adds to our knowledge of factors likely to encourage service users’ continued involvement with social prescribing interventions and highlights potential ‘threats’ to involvement. This study also highlights the importance of social prescribing as a long-term intervention and the central role of the link worker in helping service users navigate and access sources of community support.

## Abbreviations

LTC: Long-term condition
NHS: National health service
WtW: “Ways to Wellness” link worker social prescribing service
